# Two abscission zones proximal to *Lansium domesticum* fruit: one more sensitive to exogenous ethylene than the other

**DOI:** 10.3389/fpls.2015.00264

**Published:** 2015-04-21

**Authors:** Prapinporn Taesakul, Jingtair Siriphanich, Wouter G. van Doorn

**Affiliations:** ^1^Department of Horticulture, Faculty of Agriculture at Kamphaeng Saen, Kasetsart UniversityNakhon Pathom, Thailand; ^2^Postharvest Technology Innovation Center, Commission of Higher EducationBangkok, Thailand; ^3^Mann Laboratory, Department of Plant Sciences, University of California, DavisDavis, CA, USA

**Keywords:** abscission, ethylene, fruit drop, *Lansium domesticum*, longkong, 1-methylcyclopropene, ultrastructure

## Abstract

Longkong (*Lansium domesticum*) fruit grows in bunches and is also sold as bunches. Individual fruit can separate from the bunch both before and after commercial harvest. The fruit has two separation sites. The first is located between bracts on the stem and the fused sepals (separation zone 1: SZ1) and the second between the fused sepals and the fruit (separation zone 2: SZ2). True abscission occurred at both zones. We investigated whether the two zones were active at different stages of development and if they were differentially sensitive to ethylene. Abscission occurred in the SZ1 in very young fruit (fruit still at the ovary stage), during early fruit development (5 weeks after full bloom; WAFB), and in ripe and overripe fruit (15–17 WAFB). Abscission did not spontaneously occur in the SZ2, but by the time the fruit was fully ripe, 15 WAFB, and later, a slight mechanical force was sufficient to break this zone. In fruit bunches severed from the tree at 5, 8, and 13 WAFB, break strength (BS) in SZ1 decreased much more after exogenous ethylene treatment than that in SZ2. Ethylene induced abscission in the SZ1, but not in SZ2. At 5, 8, and 13 WAFB, treatment with 1-methylcyclopropane (1-MCP; an inhibitor of ethylene perception) had a small effect on BS in the SZ1 and no effect in the SZ2. It is concluded that abscission in the SZ1 was much more sensitive to ethylene than that in the SZ2. In intact plants SZ1 reacts to endogenous ethylene, e.g., as a result of stress, while SZ2 apparently allows animals to remove the ripe fruit from the tree with minimal force.

## Introduction

Longkong (*Lansium domesticum* Correa) is a tropical fruit of the Meliaceae family. The pulp of longkong is consumed fresh. The peel contains oleoresin, which is used to counter diarrhea, while the crushed seeds are used to cure fever. The fruit is widespread throughout South East Asia (Yaacob and Bamroongrugsa, 1992; Paull, 2004). Currently longkong is only traded within the South East Asian region. Shipping the fruit to distant markets must be done by air, to reduce the problem of fruit abscission.

There has been some discussion about nomenclature, e.g., whether *Lansium domesticum* is synonym with *Aglaia dookoo*. Muellner et al. (2005) separated *Aglaia* from *Lansium* whereby the former has 1–3 ovary locules, while *Lansium* has five. Fruit called langsat is also *L. domesticum*, but has a thinner fruit peel than longkong. Two fruit called duku and duku-langsat, are also *L. domesticum*; with a morphology similar to that of longkong and langsat (Song et al., 2000). Lim (2012) gave a detailed description of the various fruit in the langsat-longkong group.

Longkong fruit are berries which develop on inflorescences, bunches contain about 25–40 fruit. The ripe fruit is round, about 3–5 cm in diameter. When immature, the peel is green and the fruit pulp is white. When ripe, the peel turns yellow and the pulp becomes translucent with a juicy texture and a typical smell. The fruit pulp is then sweet with a slightly sour tone (Paull, 2004).

Longkong fruit is non-climacteric, i.e., it does not produce a peak in respiration rate or in ethylene production, and fruit ripening is not sensitive to exogenous ethylene. Normally, the fruit is harvested before full maturity. If sold on local markets, recommended harvesting is about 15 weeks after full bloom (WAFB) (Lichanporn et al., 2009). When intended for markets at greater distances, the fruit should be harvested at 13 WAFB. Fruit harvested at 15 WAFB shows a high percentage of fruit abscission (Taesakul et al., 2012).

A few species reportedly contain more than one fruit abscission zone. In citrus, abscission zones were found at the base of the pedicel, and between the sepals and the fruit. The pedicel is defined as a stem that attaches single flowers/fruit to a bigger stem of the inflorescence/fruit bunch (The stem or branch that holds a group of pedicels is called a peduncle). After exposure to ethylene, increased cell wall degrading enzyme activity was found in both abscission zones (Goren, 1993a,b; Burns, 1997). Tomato fruit also has two abscission zones (Biain de Elizalde, 1980), while peach has three (Rascio et al., 1985).

Longkong has sessile fruit, hence the pedicel is absent. The stem branches of the inflorescence/fruit bunch are called peduncles. A separation zone was found between the peduncle and the sepals (SZ1) and a second between the sepals and the fruit (SZ2; Taesakul et al., 2012). Preliminary experiments showed that in the absence of exogenous ethylene, drop of mature fruit occurred when using a small mechanical force, at the SZ2. When exogenous ethylene was present, fruit drop occurred solely at the SZ1.

We studied the separation process at the two sites in more detail. We describe anatomical changes during flower and fruit development, using light and electron microscopy and measured the break strength (BS) at the separation zones. Both ethylene and 1-MCP (which inhibits ethylene perception) were applied to test the role of ethylene in this process.

## Materials and methods

### Plant material

Bunches of longkong fruit (*Lansium domesticum* Corr.) were severed from 5 to 10 randomly selected trees, which were 12 years old and had similar canopy size and apparent health. The trees grew in an orchard in Wang Saem District of Chanthaburi Province (Thailand). One bunch was randomly severed per tree every 2 weeks, starting 3 WAFB and ending 17 WAFB. The means of average daily temperature and relative humidity during the 4 months fruit growth period were 28 ± 2.5°C and 85 ± 10%, respectively (Figure S1). Bunches were placed in perforated plastic crates (36 × 60 × 33 cm) lined with paper, and were held at ambient temperature for 1–3 h. Crates were then transported to the laboratory, using an air-conditioned van at 25–27°C. Transport took 5 h. Upon arrival the fruit was held dry in the crates, at 24–26°C for about 3 h until use.

### Physical properties during fruit development

In 10 fruit from each of the ten bunches we determined fresh weight, length, diameter of the fruit, and diameter of the fused sepals, using a digital scale (TANITA 1579, Tokyo, Japan) for weight, and Vernier calipers (Mitutoyo 532-121, Kanagawa, Japan) for length.

### Flower structure and the presence of abscission zones in the flowers

An Olympus 302990 stereo-microscope (Tokyo, Japan) with two Olympus TGHM lateral light sources were used to assess flower structure and the presence of abscission zones in the flowers. Pictures were recorded using a Dinoeye AM423X digital eyepiece (Taipei, Taiwan ROC) connected to a computer.

### Abscission zone morphology and ultrastructure

Longkong bunches were severed from the trees at 3, 8, and 13 WAFB and separated into two groups. One group was evaluated using light microscopy, the other using scanning electron microscopy.

#### Cell size

The two separation zones were removed by cutting, using a sharp razor blade. A small part of the peduncle remained attached at the proximal side of the SZ1. A small part of the fruit remained attached at the distal side of the SZ2. The isolated parts were then cut in the middle between SZ1 and SZ2. The segments were cut longitudinally, fixed in 3.7% (v/v) formaldehyde, 50% (v/v) ethanol, and 5% (v/v) acetic acid (FAA) solution, embedded in paraffin, cut into 16 μm thick slices, stained with Fast-green and Safranin-O and mounted on glass slides for observation using light microscopy (Axiostar plus, Carl Zeiss, Aalen, Germany). Cell sizes in the peduncle at the proximal side of the SZ1, in the SZ1, in the SZ2, and at the distal side of the SZ2 were determined using an ocular micrometer. Three samples (replicates) were used per treatment; nine cells were measured in each sample.

#### Ultrastructure, ethylene treatment

Eighteen longkong bunches were divided into two groups. One group was used as control. The second group was exposed to 1 μL L^−1^ ethylene for 12 h. After the ethylene treatment, the fruit were forcefully removed from the fused sepals. The sepals were also forcefully removed from the peduncle. The tissues near the separation site were collected and were dipped immediately in 2% glutaraldehyde in 0.05 M sodium phosphate buffer overnight, then treated for 2 h with 2% osmium tetroxide. The samples were dehydrated in an ethanol series, and were critical-point dried with CO_2_, and coated with 60 nm of gold/palladium. Observations were made using scanning electron microscopy (JEOL JSM-S410LV, Tokyo, Japan). Three samples were used for each stage.

### Break strength and abscission

#### During fruit development on the tree

The number of abscised fruit was determined both during development on the tree and after the harvest of almost ripe fruit. Abscission and BS were observed in bunches severed from the trees 3–17 WAFB. Abscission from the trees was low, as indicated by the low number of abscission zone scars. Abscission was determined by the drop of fruit during transportation from the trees to the laboratory. BS was determined using a push-pull force gauge (John Chatillon and Sons, Greensboro, NC), following the method of Gersch et al. (1998). To determine the BS at SZ1, the peduncle was attached to a drill clip which was mounted to the end of the push-pull force gauge. For SZ2 BS, the peduncle was removed using a razor blade. The fused sepals were attached to the drill clip. BS was determined by slowly pulling the sepal or the fruit, straight from the abscission planes, to separate them from the peduncle or the sepal. Care was taken not to pull the fruit at an angle under the abscission plane. The number of replicates was 25, consisting of five bunches (each cut from a different tree) and five fruit per bunch, at each sampling time.

#### During fruit storage

Longkong bunches were severed from five trees at 5, 8, and 13 WAFB, and brought to the laboratory. Bunches were divided into three groups. The first group was used as the control, the second group was exposed to 1 μL L^−1^ ethylene for 12 h and the third was treated with 1 μL L^−1^ 1-MCP (Ethylbloc, Floralife Inc., Walterboro, SC) for 6 h. Treatments occurred at 25°C. Fruit was then stored at 25°C for 4 days. The BS and fruit drop at SZ1 and SZ2 were determined at intervals, as described. The number of replicates was 25, consisting of five bunches (each cut from a different tree) and five fruit per bunch, at each sampling time.

### Statistical analysis

Analysis of variance was performed on all data. Mean separation was carried out using paired *t*-test and Duncan's multiple range test (SPSS 15 program, Landau and Everitt, 2004). Differences at *P* < 0.05 were considered to be significant.

## Results

### Abscission of young ovaries

**Figure 1:**
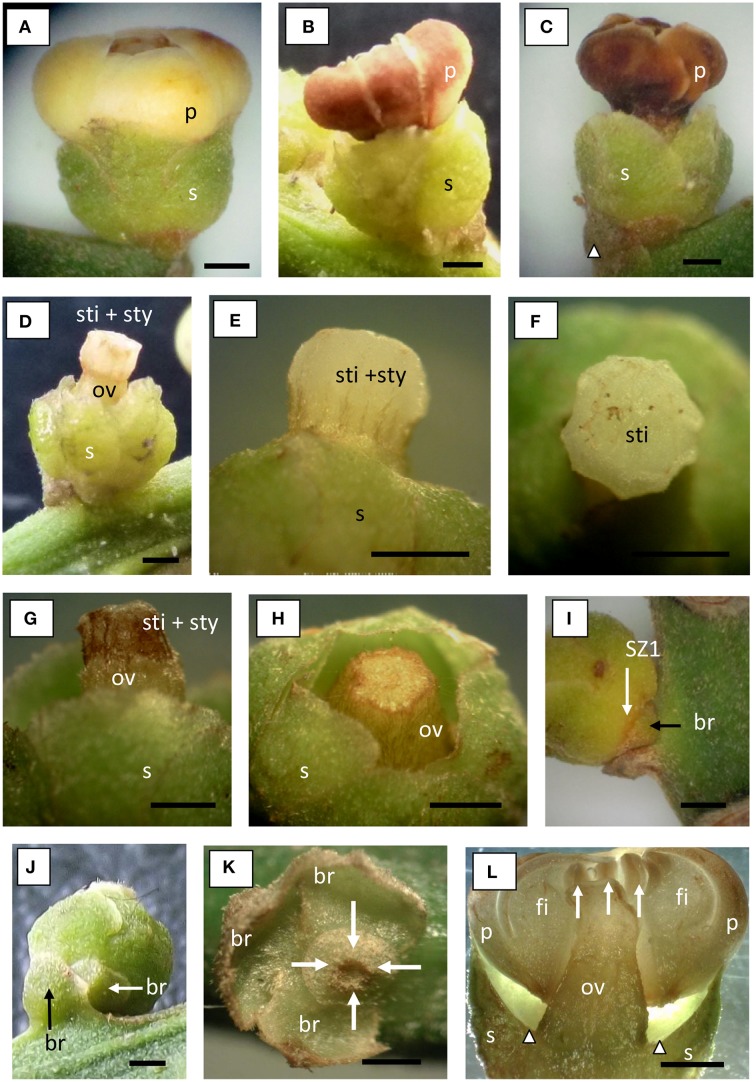
**Abscission of floral parts, including young ovaries, in longkong (*Lansium domesticum*).** **(A)** Longkong flower with fused petals (p) above the sepals (s) at opening stage; **(B)** Flower at senescence stage, the fused petals had already abscised from the fused sepals; **(C)** Flower when the fused petals had desiccated but remained on the flower; **(D)** Flower when the fused petals had already fallen off, exposing the ovary (ov), style (sty), and stigma (sti); **(E)** Close up of the style and stigma; **(F)** Top view of the stigma; **(G)** Desiccation of the stigma; **(H)** Ovary after stigma abscission; **(I)** Base of the flower showing one bract (br) and the SZ1; **(J)** Young fruit on top of 2 bracts; **(K)** After flower abscission, showing the remaining 3 bracts, arrows point at SZ1; **(L)** Longitudinal section of a flower showing the separation of the fused petals from the base of the ovary, arrows indicate anthers, arrowheads show the abscission zone of the petals and the fused stamens (fi, filament). Note abscission of (1) petals and attached ring of stamens, (2) of the stigma plus short style, and (3) the ovaries plus sepals. See text for explanation. Bars in all pictures equal 1 mm.

**Figure 2:**
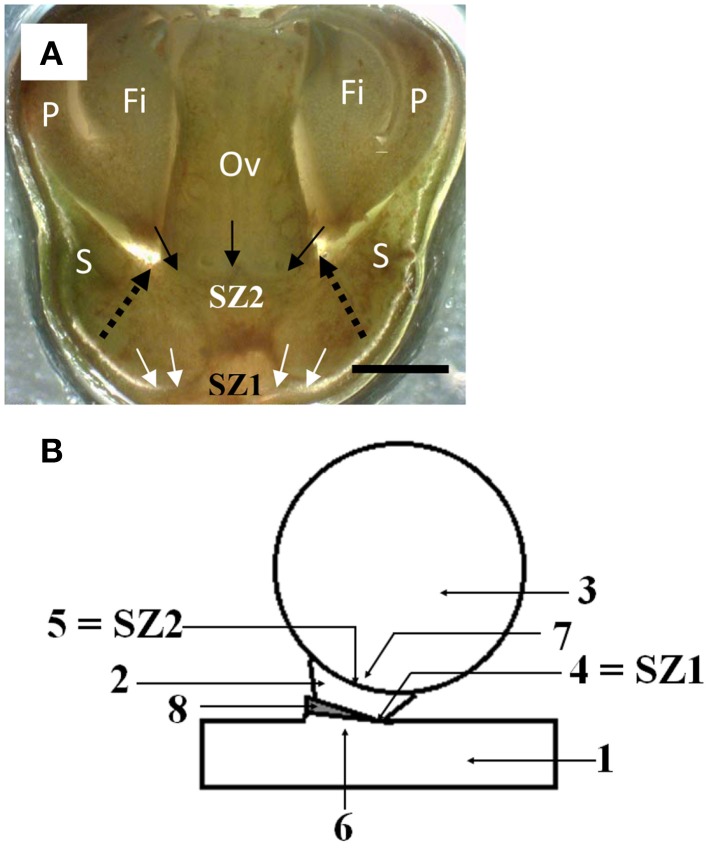
**The position of the two abscission zones in longkong fruit (*Lansium domesticum*).** **(A)** Flowers at a stage just after separation of the petals and attached stamens. The SZ1 is indicated by white arrows. It will produce separation of the ovary with the subtending, attached sepals. The future SZ2 (not yet active at this stage of development) is indicated by downward pointing arrows, and the word SZ2. The separation (abscission) zone of the petal base, indicated by dashed arrows, apparently is an extension (outer ring) of the future SZ2. Key: as in Figure 1, bar equals 1 mm. **(B)** Scheme of the abscission zones in mature fruit. Key: 1, peduncle; 2, fused sepals (calyx); 3, fruit; 4, SZ1; 5, SZ2; 6, area proximal to SZ1; 7, area distal to SZ2; 8, bract.

In the field many young ovaries were found to abscise. Figures 1, 2A show the position of the ovary, and the structure of the flower. The flower had five fleshy petals, fused at their base (Figures 1A–C,L, 2A). The petals of flowers that had just opened were off-white with creamy shades (Figure 1A). Fused with the inner base of the petals was a ring of very thick, fused filaments (Figures 1L, 2A), bearing ten anthers (Figure 1L, white arrows). Subtending the petals were five green sepals, which are also fused (Figures 1A–H,L, 2A). The sepals were distal to three bracts (Figures 1C,I–K).

The following stages of flower development were distinguished: (a) flower opening, (b) petals plus stamens separate from the flower, (c) stigma separates from the flower, (d) ovary or young fruit may separate from the flower. In Figures 1L, 2A the petals plus stamens had separated from the rest of the flower, but had not yet fallen off. In Figure 1D the petals and fused stamens had fallen. An apparent abscission zone at the petal base is indicated by arrowheads in Figure 1L. Initially, separated petals plus stamens can be removed by little mechanical force, as they were still surrounded by the sepals (Figures 1D,L, 2A). The petals were still whitish by the time of their separation. If the separated petals and stamens did not fall early (thus remain kept in check by the sepals), they showed discoloration to brown (Figure 1B), followed by desiccation (Figure 1C). After the fall of the petals plus stamens the female reproductive organs were visible (Figures 1D–H). The top of the stigma was flat, somewhat irregularly shaped at the edges (Figures 1D,F). The style was short and thick. The stigma + style separated at their junction with the hypogynous ovary. It usually fell off after slight discoloration to brown (Figures 1G,H). The ovary was situated on top of, and was fused with, the sepals (Figures 1D–H,L, 2A). This entire structure (ovary and sepals) could abscise, whereby the abscission zone was localized at the proximal side of the sepals, which was the distal side of the three subtending bracts (Figures 1I–K). The localization of this abscission zone (SZ1), after abscission, is indicated in Figures 1I,K. The three bracts are indicated and the abscission area is shown by arrows. It should be noted that the flower as well as the fruit were situated under an angle less than 90° with respect to the peduncle (Figures 1I,J, 2B).

### Position of the two separation zones of the fruit and fruit development

Abscission of ovaries (very young fruit) occurs at SZ1; it usually takes place in less than 5% of the ovaries on a bunch. The ovaries abscise together with the subtending sepals. A longitudinal section of SZ1 and the future separation zone SZ2 are indicated in Figure 2A. SZ1 is located just below the ovary, but had apparently not become fully developed by the time of ovary abscission. SZ2 extends, at its circumference, into the separation zone of the petals plus stamens. Thus, the SZ1 was located between the bracts on the peduncle and the fused sepals, while the SZ2 was between the sepals and the fruit. Figure 2B gives a scheme of the position of the two separation zones in full-grown fruit. As the fruit is sessile (has no pedicel), only the sepals separate the ovary from the distal part of the bracts.

**Figure 3:**
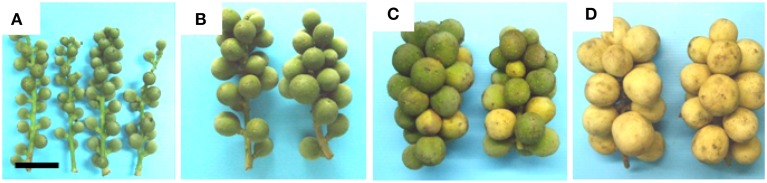
**Morphology of longkong fruit (*Lansium domesticum*) bunches.** **(A)** 5 WAFB, **(B)** 7 WAFB, **(C)** 10 WAFB, **(D)** 13 WAFB. Color break occurred at 10 WAFB. Bar in **(A)** is 5 cm. The bar also applies to **(B–D)**.

The length and diameter of longkong fruit increased almost linearly from 3 to 15 WAFB, while fruit weight increased exponentially. The diameter of the fused sepals (0.4 cm by week 3 after full bloom) only slightly increased during this period (Table S1). Fruit development included a change of the peel from green to yellow by about 10 WAFB (Figure 3).

### Cell size in and ultrastructure of the separation zones

#### Cell size

At 3 WAFB, the cells in the peduncle above the SZ1 were about 32 μm long. Cell size increased to 44 μm by 13 WAFB. At 3 WAFB the cells in SZ1 were considerably smaller (20 μm) than in those in the peduncle. Their size had increased to about 30 μm by 13 WAFB. In SZ2 the cells were about the same size as in SZ1 (Table S2).

#### Ultrastructure

**Figure 4:**
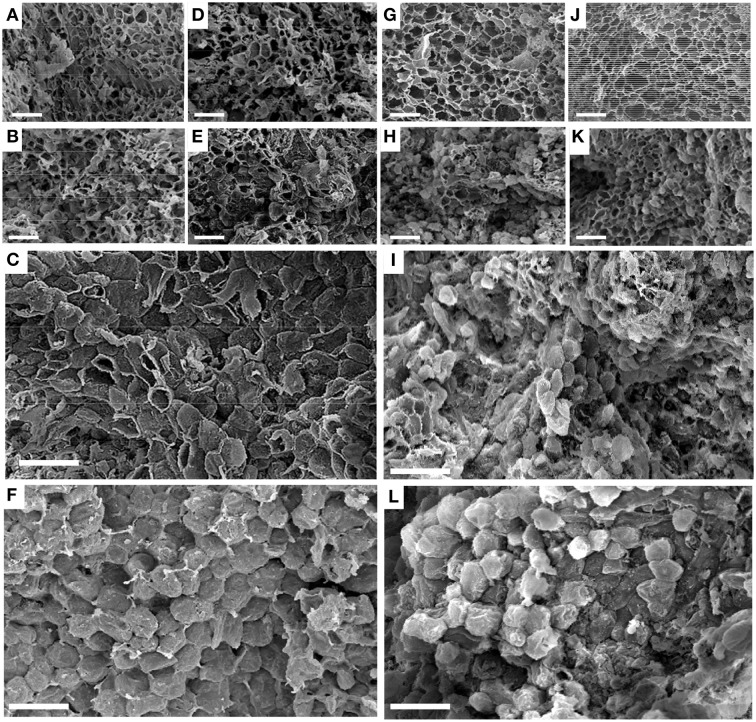
**Scanning electron micrographs of cells in longkong fruit (*Lansium domesticum*).** **(A–F)** Morphology of the SZ1 after the fruit had been forcefully removed from the peduncle. **(G–L)** Morphology of the SZ2 after the fruit had been forcefully removed from the fused sepals, either without **(A–C,G–I)** or after **(D–F,J–L)** 1 μL L^−1^ ethylene for 12 h. Times of harvest were 3 **(A,D,G,J)**, 8 **(B,E,H,K)**, and 13 **(C,F,I,L)** WAFB. Bars in all pictures equal 100 μm.

All cells in SZ1 at 3 and 8 WAFB had ruptured when the fruit together with the fused sepals (calyx) were pulled off the bunch. At these periods rupture occurred irrespective of ethylene treatment (Figures 4A,B,D,E). By contrast, at 13 WAFB and no ethylene treatment, half of the cells had separated (Figure 4C). After ethylene treatment at 13 WAFB all cells showed smooth separation (Figure 4F).

At 3 WAFB all cells in the SZ2 had ruptured when the fruit were pulled from the fused sepals, irrespective of ethylene treatment (Figures 4G,J). At 8 WAFB about half of the cells in the SZ2 showed smooth separation, while the remainder of the cells ruptured (Figures 4H,K). This was observed irrespective of ethylene exposure. At 13 WAFB, most of the cells had smoothly separated in the control (Figure 4I). All cells smoothly separated after ethylene treatment (Figure 4L).

### Fruit drop and BS in intact plants

**Figure 5:**
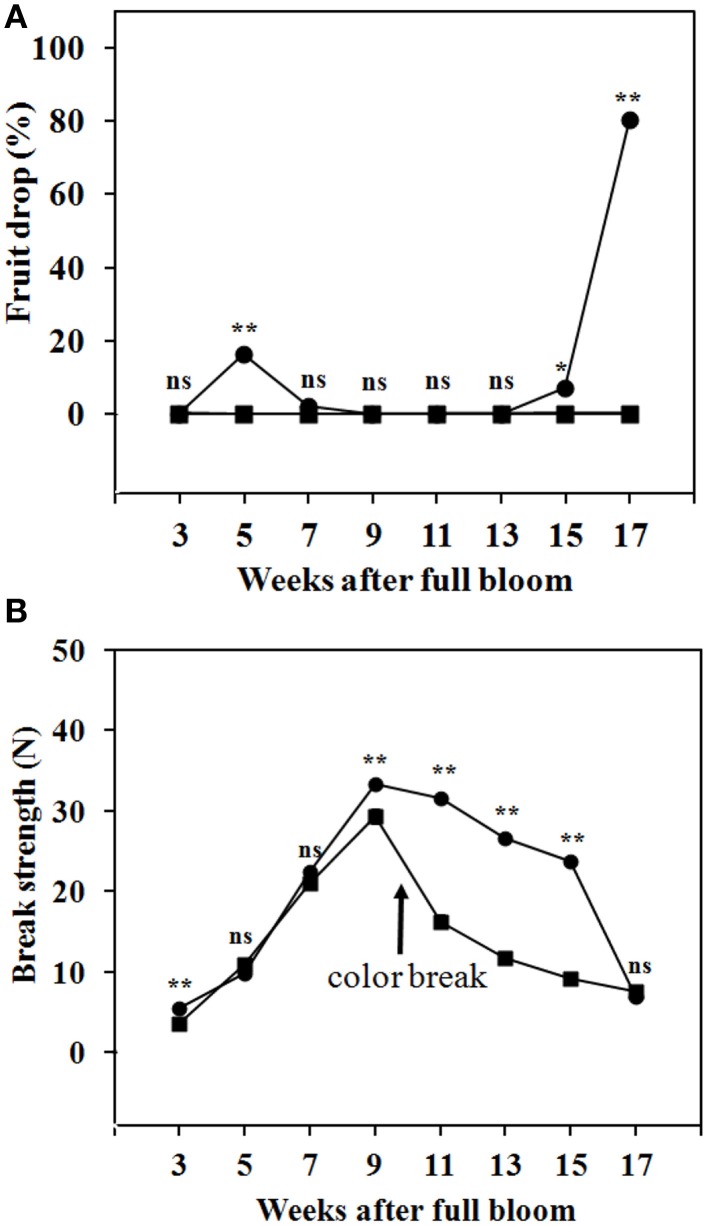
**Fruit drop and BS of longkong fruit (*Lansium domesticum*) during development on the tree. (A)** Fruit drop at the SZ1 (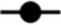) and the SZ2 (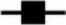). **(B)** BS at the SZ1 (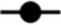) and SZ2 (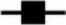). It should be noted that in these tests no mechanical force was exerted on the fruit. A slight mechanical force (about 10 N) will lead to drop by breakage at the SZ2, from 3-17 WAFB onward. Data are means of 25 replicates. *, ^**^ Significant difference at 0.05 and 0.01 respectively, ns not significant using paired *t*-test.

By 3 WAFB and later, fruit abscission from the trees was low, as only a small number of abscission zone scars were observed. Abscission was determined by the drop of fruit during transportation from the trees to the laboratory. Figure 5A shows that, in the season investigated, fruit drop at the SZ1 took place during 5–7 WAFB (immature fruit with green peel) and during 15–17 WAFB (mature with full yellow peel). No fruit drop was found at the SZ2 during fruit development on the trees (Figure 5A). The BS at SZ1 and SZ2 increased from 3 to 9 WAFB (Figure 5B). The BS at SZ1 slightly decreased from 9 to 15 WAFB (Figure 5B). By contrast, the BS at SZ2 decreased sharply from 9 to 15 WAFB (Figure 5B).

### Effects of ethylene and 1-MCP

**Figure 6:**
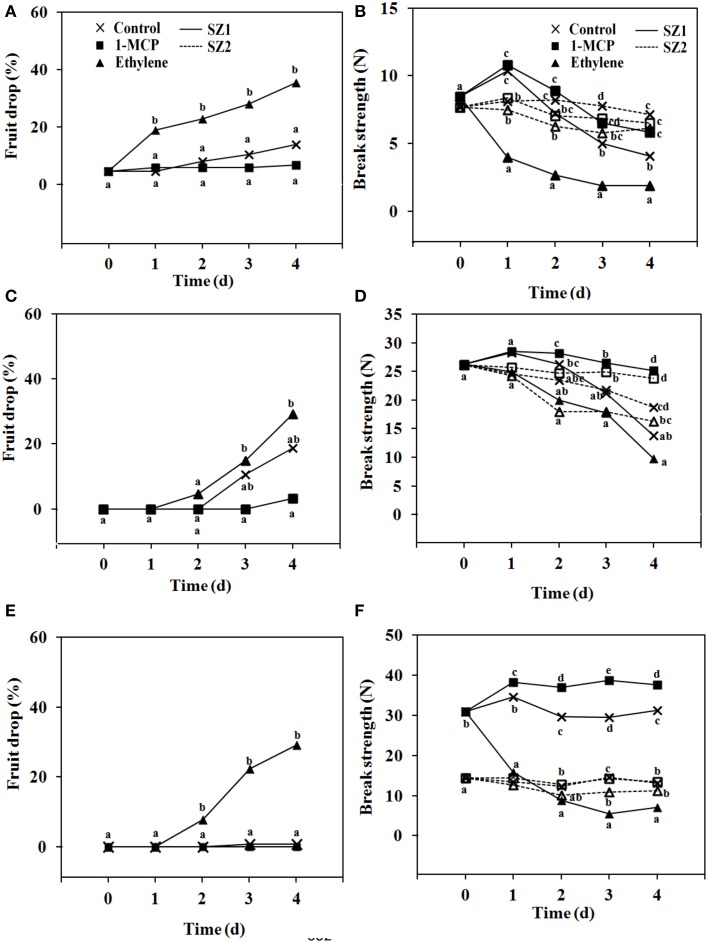
**Fruit drop and BS of longkong fruit (*Lansium domesticum*).** **(A,C,E)** Fruit drop and **(B,D,E)** BS after harvest at 5 **(A,B)**, 8 **(C,D)**, and 13 **(E,F)** WAFB. Fruit drop refers to the SZ1 after treatment with air (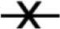), 1 μL L^−1^ 1-MCP for 6 h (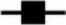) or 1 μL L^−1^ ethylene for 12 h (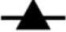), and storage at 25°C for 4 days. No fruit drop was found at the SZ2, at 5, 8, or 13 WAFB. BS refers to the SZ1 (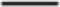) and the SZ2 (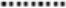) of fruit treated (▲, △) or not treated (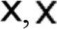) with 1 μL L^−1^ ethylene for 12 h, or treated with 1 μL L^−1^ 1-MCP (◼, ◽) for 6 h. Fruit was stored at 25°C for 4 days. Both with regard to fruit drop and BS only minor further changes were found between day 4 and 7. Note that the data on the SZ2 of day 13 WAFB of the control and the treatment with 1-MCP overlap. Data are means of 25 replicates. Data on the same day with the same letter are not different significant at *P* = 0.05, DMRT.

Effects of ethylene and 1-MCP were determined using bunches that were severed from the trees at 5, 8, and 13 WAFB. Bunches picked at 5 WAFB, and kept at 25°C, fruit drop was low in untreated controls, higher after ethylene treatment, and lower than the controls in the 1-MCP treatment (Figure 6A). In bunches severed at 8 WAFB, and kept at 25°C, fruit drop in the controls was 20% by day 4. After ethylene treatment it was about 30% by day 4 (Figure 6C). When bunches were taken at 13 WAFB, and kept at 25°C, fruit drop in the controls was absent by day 4 (Figure 6E), and was still very low by day 7 (data not shown). In fruit exposed to 1 μL L^−1^ ethylene, fruit drop was more than 20% by day 4 (Figure 6E), and more than 40% by day 7 (data not shown), while 1-MCP treatment had no effect (Figure 6E). At any of the picking dates no spontaneous fruit drop during storage was found at the SZ2, in controls, ethylene treatment, and 1-MCP treatment (data not shown).

BS was determined at 5, 8, and 13 WAFB. Each ethylene treatment resulted in a rapid drop in BS at the SZ1 (Figures 6B,D,E). By contrast, ethylene had only a slight effect at SZ2 (Figures 6B,D,E). Treatment with 1-MCP (carried out on bunches severed at 5 and 13 WAFB) had little effect, both at SZ1 and SZ2 (Figures 6B,D,E).

## Discussion

Several separation zones were observed in the flowers and fruits of longkong: one at the base of the style, one at the base of the petals and attached stamens, one between the fused sepals and three bracts on the peduncle (SZ1), and one between the sepals and the fruit (SZ2). Separation of the petals and stamens occurred together. This was apparently due to true abscission as small cells were observed, but the separation process was not studied in detail hence it cannot be excluded that the tissue was torn. Tearing off instead of abscission has been described in petals, stamens, and styles in some species. This tearing is due to the mechanical forces generated by the growing fruit (Reiche, 1885; Wacker, 1911; van Doorn and Stead, 1997).

SZ1 and SZ2 of longkong fruit contained cells that were much smaller than the adjacent cells. Abscission zones usually contain small cells, but the presence of small cells is by itself inadequate proof of true abscission. After fruit drop at SZ1 (either without exogenous ethylene or after ethylene treatment) the zone showed smooth separation rather than cell breakage. This strongly suggests that SZ1 is a true abscission zone. After slight mechanical force is exerted on ripe longkong fruit, this leads to fruit drop at SZ2. In addition, most cells were easily separated, strongly indicating that an abscission process was in progress. Nonetheless, as the fruit did not abscise spontaneously, a number of cells apparently still needed to be broken. It was previously suggested that there was no true abscission zone at SZ2 (Taesakul et al., 2012). This is correct insofar the zone did not show spontaneous abscission, but at the same time not true because most of the zone cells showed smooth cell walls (Figure 4L) after separating the fruit from the bunch at 13 WAFB. The data show that middle lamella separation (abscission) occurred in many cells at SZ2.

In many abscission zones the vascular bundle is the only part not undergoing wall separation (Roberts et al., 2002), but this does not hamper abscission. This may also be true for SZ1. However, because of the lack of spontaneous abscission even by 15 WAFB, SZ2 seems to contain more cells that have not undergone cell separation than SZ1 by the time of abscission at that zone.

During fruit development on the trees, the BS at SZ1, at 15 WAFB, was clearly higher than SZ2, but fruit drop and abscission was higher at SZ1 (Figure 5). The results seem conflicting, but they could be explained either by tearing off of the fruit due to growth, or by normal abscission. We found smooth abscission scars, which seems to exclude tearing off of the fruit. The explanation of the apparent discrepancy may possibly be that a subset of the fruit was attacked by pests such as fungi, causing a local increase in ethylene production and thus abscission. This fruit then dropped, hence their BS must have been close to zero. However, the majority of the fruit that remained was healthy, thus had high BS. This means that the average BS, determined in the remaining fruit, was high. However, one may ask why fruit dropped at SZ1 and not at SZ2, while both zones were equally exposed to fungal-induced ethylene. The explanation might be that SZ2 is less ethylene-sensitive. The difference in ethylene sensitivity between SZ1 and SZ2 is shown in Figure 6, where harvested fruit was exposed to ethylene.

After ethylene treatment of fruit harvested at the harvest time for local consumption (i.e., 15 WAFB) the BS at SZ1 had decreased significantly. The cells that had separated in the SZ1 showed smooth cell walls, indicative of a natural abscission processes. These data indicate that SZ1 contains an abscission zone that is normally sensitive to ethylene, similar to the zones in many other fruit, for example tomato (Tabuchi et al., 2000).

The anatomical data on longkong separation zones are similar to those in citrus (Goren, 1993a,b). One citrus fruit abscission zone (called A) is located at the proximal part of the pedicel, which is similar to the location of SZ1 in longkong. A more distal abscission zone in citrus is located between the sepals (calyx) and the fruit (called zone C), which is similar to the position of the SZ2 in longkong. The main difference between longkong and citrus is the closer proximity of the two abscission zones in longkong, as longkong has no pedicel. In longkong the two abscission zones are located at both sides (distal and proximal) of the fused sepals. Similarly, tomato has two abscission zones in the fruit stem. A proximal zone is located between the pedicel and peduncle (comparable to SZ1), and a distal zone between fruit and pedicel (slightly comparable to SZ2; Biain de Elizalde, 1980). In peach fruit stems three abscission zones have been found, the most distal one is at the junction of the fruit with the pedicel (slightly similar to SZ2 in longkong), and two others are in the pedicel (Rascio et al., 1985).

During growth on the trees, ovary and fruit drop occurs during three periods. Very young fruit (ovaries) abscised just after the petal life span had terminated. Young fruitlets abscised about 5 WAFB. Mature fruit abscised at about 15 WAFB. Similar abscission periods have been described for several other fruit. In *Citrus lemon*, for example, a period of massive abscission results in loss of flowers and ovaries. About a week later, fruit having a diameter similar to that of full-grown peas abscised. The third period of abscission occurred in the spring, reducing the number of growing fruitlets, which were by then about the size of a golf ball. This is similar to what is called June fruit drop in many species in the Northern Hemisphere (Goren, 1993a,b; Iglesias et al., 2006). Citrus fruit remained on the tree until over-mature, but seems to eventually abscise.

Flower abscission and early fruit abscission is usually not determined by the absence of pollination/fertilization but by competition between pollinated flowers and between fertilized ovaries. Abscission of fertilized flowers and very young fruit was found to be regulated by the availability, to the organ, of mineral nutrients or carbohydrates (Stephenson, 1981; Racskó et al., 2006; McFadyen et al., 2012). Abscission of the ovaries in longkong also does not seem related to the absence of fertilization, as longkong fruit is thought to be predominantly parthenocarpic (Bernardo et al., 1961). Ovary abscission could possibly therefore relate to competition. Similarly, it could be suggested that abscission of longkong fruit at the immature stage is mainly due to competition (Stephenson, 1981; Wright, 1989; Iglesias et al., 2006).

The longkong ovaries are fused with the subtending sepals. Ovaries therefore abscise together with the sepals, at an abscission zone that is located between the sepals and three subtending bracts (thus at SZ1). We found that (a) abscission of ovaries in longkong flowers occurred only at SZ1, (b) abscission of immature fruit by 5 WAFB took place only at SZ1, and (c) spontaneous abscission of ripe fruit (not the fall after slight mechanical force) was also restricted to SZ1. Only after a slight mechanical force was exerted on ripe (mature) fruit, was abscission found at SZ2. This is quite different from citrus, where abscission of the ovaries and that of very small fruit, i.e., during 6–8 weeks after fruit set, took place exclusively at abscission zone A (similar to SZ1), while by the time of the June abscission period abscission zone A had become progressively inactivated while abscission zone C (similar to SZ2) began to operate and later on all abscission took place at abscission zone C. Only during 2–3 intermediate weeks is abscission found at both sites (Goren, 1993a,b; Iglesias et al., 2006). The data on longkong also differ from those in tomato and peach. Biain de Elizalde (1980) reported that small immature tomato fruit abscised at the proximal zone (similar to SZ1) while ripe fruit showed abscission at the distal zone (slightly similar to SZ2). In peach Rascio et al. (1985) found that the abscission zone at the junction of the fruit and the pedicel (similar to SZ1) is responsible for abscission of floral buds, open flowers, and very young fruit. Two abscission zones in the pedicel become activated later on, one during early June drop, the other during late June drop.

At 5, 8, and 13 WAFB we observed increased abscission at SZ1 after ethylene treatment while SZ2 did not respond to ethylene by increasing abscission. Ethylene only induced a small decrease in the BS at SZ2 (where abscission of mature fruit took place, after slight mechanical force only), but did not induce abscission at SZ2. These data show that SZ1 was highly sensitive to ethylene, whereas SZ2 was only slightly sensitive, not enough to induce abscission. This is quite different from tomato fruit where ethephon (ethylene) treatment increased the abscission of ripe fruit but not of small immature fruit (Biain de Elizalde, 1980). In citrus ethylene increased abscission in both abscission zone A (although only until week 6–8 after fruit set, and no longer thereafter) and abscission zone C (Goren, 1993b; Iglesias et al., 2006). This is also different from our findings in longkong fruit.

In citrus it depends on the developmental stage of zone A whether ethylene induces abscission in this zone or not (Goren, 1993b; Iglesias et al., 2006). The present data on longkong show that exogenous ethylene was also not able to induce abscission in the SZ2, even though the BS became slightly lower after the ethylene treatment. SZ2 was considerably less sensitive to exogenous ethylene than the SZ1. It is therefore possible that the SZ2 is relatively insensitive to endogenous ethylene. This needs to be determined in further experiments.

After a short transportation period (from the field to the laboratory) of bunches harvested at 13–15 WAFB, fruit drop during transportation was low. In the presen*t*-tests (Figure 6), which lasted only 4 days, no fruit drop was found in controls harvested at 13 WAFB. By contrast, in other (unpublished) postharvest storage simulation experiments in the laboratory, using fruit harvested at 13 WAFB, abscission mainly increased after day 4 of storage, and varied between and 10 and 70% by day 10. Drop occurred mostly at SZ1 with about 5% at SZ2. High rates of abscission might be due mainly to ethylene production of fruit infected by fungus. An additional practical test (also unpublished data) found that fruit harvested at 13 WAFB and undergoing long sea transportation in a container (7 days at 18°C) showed about 70–80% fruit drop by the end of transportation, increasing to 100% after transfer to ambient temperature for 2 days. Most of the drop (about 95%) occurred at SZ1. These data also suggested that fruit infected with fungus produced relatively high ethylene concentrations in the closed container, inducing abscission at SZ1. This abscission can largely be prevented, as it was previously reported that a 6 h treatment with 1 μL L^−1^ 1-MCP drastically reduces fruit drop at the SZ1 (Taesakul et al., 2012).

A hypothesis might be proposed as to the possible function of the two separation zones in longkong fruit. As the separation at SZ1 by 13 WAFB was significantly promoted by exogenous ethylene, this zone could possibly function in response to changes in endogenous ethylene levels or to changes in ethylene sensitivity. It could therefore be responsive to factors that cause physiological stress. Infection of longkong fruit with fungi, of which *Phomopsis* sp. and *Lasiodiplodia theobromae* are the most important (Kaewsorn and Sangchote, 2003), might induce such a stress. SZ2 was much less affected by the ethylene treatment than SZ1. Separation at SZ2 had already started before the change in peel color (Figure 6B), as indicated by the presence of smooth cell walls after pulling the fruit from the fused sepals (Figure 4H). By 15 WAFB the BS at SZ2 was quite low, although not low enough for spontaneous abscission. This low BS possibly allows animals to remove healthy and ripe fruit from the tree, using only slight mechanical force.

It is concluded that longkong ovary and fruit drop on the tree only occurred at SZ1, which contains an abscission zone that is highly sensitive to exogenous ethylene. By contrast, fruit drop at SZ2 was not induced by treatments with exogenous ethylene, even though cell separation in this zone had already started by the time of fruit coloration, and was slightly increased by the ethylene treatment. Incomplete cell separation at SZ2 seems to explain why no spontaneous fruit drop occurred at this zone. By 15 WAFB only a small mechanical force induced full separation at SZ2. The two zones are thus functionally different: one shows abscission in response to an increase in exogenous ethylene concentrations while the other does not. We suggested a hypothesis to account for the presence of two abscission zones with different characteristics.

## Author contributions

PT did the experimental work, carried out data analysis, and drafted the manuscript; WD interpreted the data and revised the manuscript; JS designed the work, acquired funding, supervised experiments, interpreted the data, and was involved in revising the manuscript. The three authors approved the final version and agree to be accountable for all aspects of the work.

### Conflict of interest statement

The authors declare that the research was conducted in the absence of any commercial or financial relationships that could be construed as a potential conflict of interest.
