# Supplementary material for: Two abscission zones proximal to Lansium domesticum fruit: one more sensitive to exogenous ethylene than the other
Source: Front Plant Sci. 2015 Apr 21;6:264. doi: 10.3389/fpls.2015.00264 (PMC4404946; doi:10.3389/fpls.2015.00264)
Supplement: Supplementary file 1 [file DataSheet1.DOCX]

**Supplementary data**

**
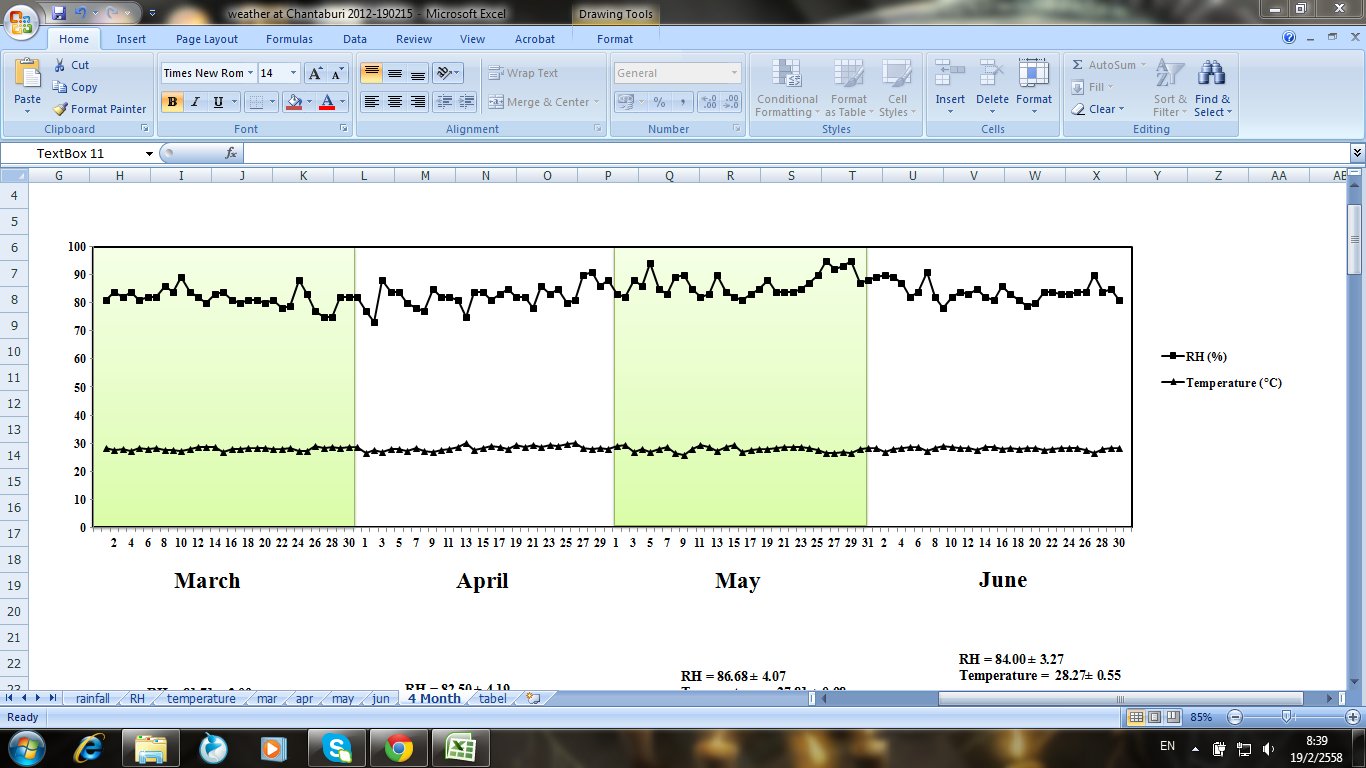
**

**Figure S1** Average daily temperature and relative humidity at Chanthaburi Horticultural Research Station in 2012

**Table S1** Weight, length and diameter of fruit and the diameter of the calyx during longkong fruit development

| **Physical properties^1^** | **Weeks after full bloom** | | | | | | |
| --- | --- | --- | --- | --- | --- | --- | --- |
|  | **3** | **5** | **7** | **9** | **11** | **13** | **15** |
| **Weight of fruit (g)** | 0.13±0.01a | 0.96±0.06a | 2.93±0.09b | 8.19±0.23c | 14.16±0.63d | 21.06±0.64e | 27.63±0.78f |
| **Length of fruit (cm)** | 0.55±0.01a | 1.22±0.03b | 1.85±0.02c | 2.57±0.03d | 3.03±0.05e | 3.43±0.04f | 4.00±0.05g |
| **Diameter of fruit (cm)** | 0.53±0.01a | 1.13±0.03ab | 1.64±0.02b | 2.32±0.03c | 2.81±0.04cd | 3.07±0.04d | 3.50±0.03e |
| **Diameter of calyx (cm)** | 0.48±0.01a | 0.50±0.01a | 0.59±0.02b | 0.62±0.00bc | 0.63±0.01bc | 0.66±0.03c | 0.72±0.01d |

^1^Data are means ± SD. Within each physical property, numbers followed by the same letter are not different at the 0.05 level of significance.

**Table S2** Size of cell in the two abscission zones of longkong fruit and in the neighboring area

| **Weeks after full bloom^1^** | **Cell location** | | | | | |
| --- | --- | --- | --- | --- | --- | --- |
|  | **Peduncle**  **(µm)** | **Calyx below SZ1 (µm)** | **Calyx above SZ2 (µm)** | **Fruit**  **(µm)** | **SZ1**  **(µm)** | **SZ2**  **(µm)** |
| **3** | 32.8±4.2b | 33.3±4.6b | 32.2±3.5b | 20.6±3.8a | 21.1±2.2a | 21.1±3.3a |
| **8** | 41.1±2.1c | 41.4±5.3c | 37.8±6.4c | 23.3±2.4a | 26.7±3.5ab | 28.9±4.2b |
| **13** | 43.9±6.3b | 45.6±6.1b | 54.4±5.1c | 40.0±6.8b | 31.1±3.3a | 33.3±4.3a |

^1^Data are means ± SD. Within each week after full bloom, numbers followed by the same letter are not different at the 0.05 level of significance.
